# Supplementary material for: Beverage Intake and the Risk of Gestational Diabetes Mellitus: The SECOST
Source: Nutrients. 2021 Jun 27;13(7):2208. doi: 10.3390/nu13072208 (PMC8308348; doi:10.3390/nu13072208)
Supplement: Supplementary file 1 [file nutrients-13-02208-s001.zip › nutrients-1250454-supplementary.pdf]

Supplementary Table S1. Energy and macronutrient intakes of women by cultured-milk drinks and fruit juice categories.

	**Cultured-milk drinks categories**					**Fruit juice categories**						
**Pre-pregnancy**	**T1 (n=47)**	**T2 (n= 73)**	**T3 (n= 30)**	**F^a**	**p-value**	**T1 (n= 70)**	**T2 (n= 97)**	**T3 (n= 118)**	**F^b**	**p-value**	**F^c**	**p-value**
	mean ± se											
Energy intake (kcal/day)	2353 ± 147.64^{a}	2092 ± 69.37^{b}	2702 ± $287.47^{a,b}$	4.15	0.02*	2011 ± 121.78^{a}	2176.90 ± 103.89^{b}	2574.68 ± $94.11^{a,b}$	7.73	0.01*	1.29	0.28
Carbohydrate (g/1000 kcal)	139.88 ± 2.66	142.25 ± 2.13	142.56 ± 3.29	2.94	0.75	134.55 ± $2.43^{a,b}$	146.20 ± 2.07^{a}	145.58 ± 1.87^{b}	8.21	0.001**	0.75	0.56
Sugar (g/1000 kcal)	40.69 ± 2.03	41.26 ± 1.62	45.41 ± 2.51	1.23	0.29	33.07 ± $2.00^{a,b}$	43.28 ± 1.71^{a}	46.62 ± 1.54^{b}	14.73	0.001**	2.62	0.05
Fat (g/1000 kcal)	22.57 ± 1.05	23.56 ± 0.84	24.43 ± 1.30	0.65	0.53	26.52 ± $0.87^{a,b}$	22.15 ± 0.74^{a}	22.64 ± 0.67^{b}	8.42	0.001**	5.40	**0.001***
Protein (g/1000 kcal)	33.81 ± 1.11^{c}	38.35 ± 0.89^{c}	35.44 ± 1.37	5.27	0.01*	38.75 ± $0.91^{a,b}$	35.61 ± $0.78^{a,c}$	32.54 ± $0.70^{b,c}$	14.76	0.001**	1.13	0.35
**First trimester**	**T1 (n=29)**	**T2 (n= 49)**	**T3 (n= 31)**	**F^a**	**p-value**	**T1 (n=63)**	**T2 (n= 77)**	**T3 (n= 117)**	**F^b**	**p-value**	**F^c**	**p-value**
Energy intake (kcal/day)	2017 ± 142.63^{a}	2089 ± 129.16^{b}	2603 ± $193.01^{a,b}$	3.85	0.02*	1890 ± 117.33^{a}	2136.34 ± 106.53^{b}	2439.08 ± $86.3^{a,b}$	7.43	0.001*	0.75	0.56
Carbohydrate (g/1000 kcal)	140.92 ± 3.86	143.39 ± 2.93	143.06 ± 3.72	0.18	0.83	135.71 ± $2.44^{a,b}$	144.18 ± 2.21^{a}	143.19 ± 1.79^{b}	4.00	0.02*	1.51	0.21
Sugar (g/1000 kcal)	43.20 ± 3.11	47.01 ± 2.36	52.05 ± 2.99	2.09	0.13	41.35 ± 1.96^{a}	45.19 ± 1.78	48.25 ± 1.44^{a}	4.05	0.02*	1.46	0.23
Fat (g/1000 kcal)	32.10 ± 1.32	30.02 ± 0.99	31.83 ± 1.27	1.04	0.36	33.16 ± 0.93	31.15 ± 0.85	31.31 ± 0.69	1.60	0.20	1.36	0.26
Protein (g/1000 kcal)	37.07 ± 1.41	36.55 ± 1.07	36.42 ± 1.36	0.06	0.94	38.93 ± $0.96^{a,b}$	35.61 ± 0.87^{a}	35.62 ± 0.71^{b}	4.51	0.01	0.31	0.87

Note. Cultured-milk drinks categories: Pre-pregnancy: T1: < 13.10 g, T2: 13.10 – 17.90 g, T3: > 17.90 g; Fruit juice categories: T1: < 35.72 g, T2: 35.72 – 71.43 g, T3: > 71.43 g); First trimester: T1: < 8.34 g, T2: 8.34 – 17.86 g, T3: > 17.86 g; Fruit juice categories: T1: < 35.72 g, T2: 35.72 – 71.43 g, T3: > 71.43 g).
F^a = F between cultured-milk drink categories; F^b= F between fruit juice categories; F^c= F between cultured-milk drink categories and fruit juice categories
Adjusted for age, education level and pre-pregnancy BMI. Similar superscript letters indicated statistically significant difference. *p<0.05

Supplementary Table S2: Food groups based on dietary patterns between T3 of cultured-milk and T3 of fruit juice drinkers

**Pre-pregnancy**	**T3 Cultured-milk drinks (> 17.90 g) (n= 30)**	Bread, fish, eggs, chicken, green leafy vegetables, milk, cereal & other cereal products, rice, other vegetables, tubers, oil, **spreads, sweet foods, tea, coffee, sugar and creamer**
	**T3 Fruit juice (> 71.43 g) (n=118)**	Bread, fish, eggs, chicken, green leafy vegetables, milk, cereal & other cereal products, rice, other vegetables, tubers, oil, **nuts, seeds & legumes, cruciferous vegetables**
**First trimester**	**T3 Cultured-milk drinks (> 17.86 g) (n= 31)**	Bread, fish, eggs, chicken, green leafy vegetables, nuts, seeds & legumes, milk, dairy products, spreads, cereal & other cereal products, **rice, processed foods, sauces, sugar and creamer**
	**T3 Fruit juice (> 71.43 g) (n= 117)**	Bread, fish, eggs, chicken, green leafy vegetables, nuts, seeds & legumes, milk, dairy products, spreads, cereal & other cereal products, **red meat, seafood, fruits, other vegetables, tubers, cruciferous vegetables**

Supplementary Table S3. Energy and nutrient intakes of women by categories of energy derived from beverages.

	Energy derived from beverages			F	p-value
**Pre-pregnancy**	**T1 (< 154 kcal) (n= 151)**	**T2 (154 – 297 kcal/day) (n= 151)**	**T3 (> 297 kcal/day) (n= 150)**		
	mean ± se				
Energy intake (kcal/day)	1821 ± 63.63	2047 ± 52.26	2611 ± 101.46	29.10	0.001*,a
Carbohydrate (g/day)	251.49 ± 9.94	293.32 ± 8.47	366.00 ± 14.14	27.19	0.001*,a
Sugar (g/day)	57.99 ± 2.90	82.38 ± 3.12	110.85 ± 4.55	54.08	0.001*,a
Fat (g/day)	41.60 ± 1.72	49.46 ± 2.07	60.10 ± 2.71	17.67	0.001*,a
Protein (g/day)	68.61 ± 2.64	74.70 ± 2.50	88.79 ± 3.94	11.18	0.001*,a
**First trimester**	**T1 (< 215 kcal/day) (n=150)**	**T2 (215 – 395 kcal/day) (n= 152)**	**T3 (> 395 kcal/day) (n= 150)**		
	mean ± se				
Energy intake (kcal/day)	1577 ± 52.76	1946 ± 50.77	2578 ± 89.26	57.53	0.001*,a
Carbohydrate (g/day)	224.45 ± 8.34	272.17 ± 7.92	356.63 ± 12.20	43.80	0.001*,a
Sugar (g/day)	56.71 ± 3.55	80.26 ± 2.53	130.58 ± 4.30	114.05	0.001*,a
Fat (g/day)	49.17 ± 2.09	63.51 ± 2.24	83.56 ± 3.08	47.27	0.001*,a
Protein (g/day)	57.49 ± 1.59	69.69 ± 2.36	99.13 ± 4.09	50.44	0.001*,a
**Second trimester**	**T1 (< 239 kcal/day) (n=151)**	**T2 (239 – 406 kcal/day) (n= 151)**	**T3 (> 406 kcal/day) (n= 150)**		
	mean ± se				
Energy intake (kcal/day)	1771 ± 46.34	2092 ± 53.74	2655 ± 91.83	44.76	0.001*,a
Carbohydrate (g/day)	252.66 ± 7.01	290.27 ± 8.45	370.14 ± 13.73	34.99	0.001*,a
Sugar (g/day)	56.86 ± 2.03	81.52 ± 2.63	125.33 ± 5.65	84.27	0.001*,a
Fat (g/day)	56.40 ± 2.07	71.62 ± 2.37	88.67 ± 3.28	37.89	0.001*,a

Protein (g/day)	61.15 ± 2.04	70.11 ± 1.84	93.99 ± 1.64	42.08	0.001*[,a]

Adjusted for age, education level and pre-pregnancy BMI

[a] Significant difference between each other, which all had statistical significance.

*$p<0.05$

References

1. Malik, V.S.; Schulze, M.B.; Hu, F.B. Intake of sugar-sweetened beverages and weight gain: A systematic review. *Am. J. Clin. Nutr.* **2006**, *84*, 274–288, doi:10.1093/ajcn/84.1.274.

2. Imamura, F.; O'Connor, L.; Ye, Z.; Mursu, J.; Hayashino, Y.; Bhupathiraju, S.N.; Forouhi, N.G. Consumption of sugar sweetened beverages, artificially sweetened beverages, and fruit juice and incidence of type 2 diabetes: Systematic review, meta-analysis, and estimation of population attributable fraction. *Br. J. Sports Med.* **2016**, *351*, h3576, doi:10.1136/bjsports-2016-h3576rep.

3. U.S. Department of Health and Human Services and U.S. Department of Agriculture Dietary Guidelines for Americans 2015 – 2020.

4. Nielsen, S.J.; Popkin, B.M. Changes in beverage intake between 1977 and 2001. *Am. J. Prev. Med.* **2004**, *27*, 205–210, doi:10.1016/j.amepre.2004.05.005.

5. Xi, B.; Huang, Y.; Reilly, K.H.; Li, S.; Zheng, R.; Barrio-Lopez, M.T.; Martinez-Gonzalez, M.A.; Zhou, D. Sugar-sweetened beverages and risk of hypertension and CVD: A dose-response meta-analysis. *Br. J. Nutr.* **2015**, *113*, 709–717, doi:10.1017/S0007114514004383.

6. Singh, G.M.; Micha, R.; Khatibzadeh, S.; Lim, S.; Ezzati, M.; Mozaffarian, D. Estimated global, regional, and national disease burdens related to sugar-sweetened beverage consumption in 2010. *Circulation* **2015**, *132*, 639–666, doi:10.1161/CIRCULATIONAHA.114.010636.

7. Keller, A.; Heitmann, B.L.; Olsen, N. Sugar-sweetened beverages, vascular risk

factors and events: A systematic literature review. *Public Health Nutr.* **2015**, *18*, 1145–1154, doi:10.1017/S1368980014002122.

8. Woodward-Lopez, G.; Kao, J.; Ritchie, L. To what extent have sweetened beverages contributed to the obesity epidemic? *Public Health Nutr.* **2011**, *14*, 499–509, doi:10.1017/S1368980010002375.
9. Liu, S.; Manson, J.A.E.; Buring, J.E.; Stampfer, M.J.; Willett, W.C.; Ridker, P.M. Relation between a diet with a high glycemic load and plasma concentrations of high-sensitivity C-reactive protein in middle-aged women. *Am. J. Clin. Nutr.* **2002**, *75*, 492–498, doi:10.1093/ajcn/75.3.492.
10. Lundeen, E.A.; Park, S.; Woo Baidal, J.A.; Sharma, A.J.; Blanck, H.M. Sugar-Sweetened Beverage Intake Among Pregnant and Non-pregnant Women of Reproductive Age. *Matern. Child Health J.* **2020**, *24*, 709–717, doi:10.1007/s10995-020-02918-2.
11. Gamba, R.J.; Leung, C.W.; Petito, L.; Abrams, B.; Laraia, B.A. Sugar sweetened beverage consumption during pregnancy is associated with lower diet quality and greater total energy intake. *PLoS One* **2019**, *14*, 1–13, doi:10.1371/journal.pone.0215686.
12. Petherick, E.S.; Goran, M.I.; Wright, J. Relationship between artificially sweetened and sugar-sweetened cola beverage consumption during pregnancy and preterm delivery in a multi-ethnic cohort: analysis of the Born in Bradford cohort study. *Eur. J. Clin. Nutr.* **2014**, *68*, 404–407, doi:10.1038/ejcn.2013.267.

13. Cohen, J.F.W.; Rifas-Shiman, S.L.; Young, J.; Oken, E. Associations of Prenatal and Child Sugar Intake With Child Cognition. *Am. J. Prev. Med.* **2018**, *54*, 727–735, doi:10.1016/j.amepre.2018.02.020.
14. Gillman, M.W.; Rifas-Shiman, S.L.; Fernandez-Barres, S.; Kleinman, K.; Taveras, E.M.; Oken, E. Beverage intake during pregnancy and childhood adiposity. *Pediatrics* **2017**, *140*, doi:10.1542/peds.2017-0031.
15. Wright, L.S.; Rifas-Shiman, S.L.; Oken, E.; Litonjua, A.A.; Gold, D.R. Prenatal and early life fructose, fructose-containing beverages, and midchildhood asthma. *Ann. Am. Thorac. Soc.* **2018**, *15*, 217–224, doi:10.1513/AnnalsATS.201707-530OC.
16. Jen, V.; Erler, N.S.; Tielemans, M.J.; Braun, K.V.E.; Jaddoe, V.W.V.; Franco, O.H.; Voortman, T. Mothers' intake of sugar-containing beverages during pregnancy and body composition of their children during childhood: The Generation R Study. *Am. J. Clin. Nutr.* **2017**, *105*, 834–841, doi:10.3945/ajcn.116.147934.
17. Chen, L.; Hu, F.B.; Yeung, E.; Willett, W.; Zhang, C. Prospective study of pre-gravid sugar-sweetened beverage consumption and the risk of gestational diabetes mellitus. *Diabetes Care* **2009**, *32*, 2236–2241, doi:10.2337/dc09-0866.
18. Ahmad, M.H. Food Consumption Patterns: Findings from the Malaysian Adults Nutrition Survey (MANS) 2014. In Proceedings of the Medical Journal of Malaysia; 2015; Vol. 70, p. 16.
19. Norimah, A.K.; Safiah, M.; Jamal, K.; Haslinda, S.; Zuhaida, H.; Rohida, S.;

Fatimah, S.; Norazlin, S.; Poh, B.K.; Kandiah, M.; et al. Food Consumption Patterns: Findings from the Malaysian Adult Nutrition Survey (MANS). *Malays. J. Nutr.* **2008**, *14*, 25–39.

20. Yong, H.Y.; Mohd Shariff, Z.; Rejali, Z.; Mohd Yusof, B.N.; Yasmin, F.; Palaniveloo, L. Seremban Cohort Study (SECOST): a prospective study of determinants and pregnancy outcomes of maternal glycaemia in Malaysia. *BMJ Open* **2018**, *8*, e018321, doi:10.1136/bmjopen-2017-018321.
21. Ministry of Health Malaysia *Perinatal Care Manual 3rd Edition*; Division of Family Health Development, MOH: Putrajaya, Malaysia, 2013;
22. Ministry of Health Malaysia *The Malaysian Adults Nutrition Survey (MANS)-Findings Report 2003*; Ministry of Health Malaysia, Malaysia: Nutrition Section Family Health Development Division, Putrajaya, 2007;
23. WHO *Physical status: The Use and Interpretation of Anthropometry. Report of a WHO Expert Committee.*; World Health Organization, Ed.; WHO Technical Report Series No. 854.: Geneva, 1995;
24. IOM *Weight Gain During Pregnancy: Reexamining the Guidelines*; Rasmussen, K.M., Yaktine, A.L., Eds.; The National Academies Press: Washington (DC), 2009; ISBN 9780309131131.
25. Drouin-Chartier, J.P.; Zheng, Y.; Li, Y.; Malik, V.; Pan, A.; Bhupathiraju, S.N.; Tobias, D.K.; Manson, J.A.E.; Willett, W.C.; Hu, F.B. Changes in consumption of sugary beverages and artificially sweetened beverages and subsequent risk of type 2 diabetes: Results from three large prospective U.S. Cohorts of women

and men. *Diabetes Care* **2019**, dc190734, doi:10.2337/dc19-0734.

26. Mathur, K.; Agrawal, R.K.; Nagpure, S.; Deshpande, D. Effect of artificial sweeteners on insulin resistance among type-2 diabetes mellitus patients. *J. Fam. Med. Prim. care* **2020**, *9*, 69–71, doi:10.4103/jfmpc.jfmpc_329_19.

27. Chen, L.; Hu, F.B.; Yeung, E.; Tobias, D.K.; Willett, W.C.; Zhang, C. Prepregnancy consumption of fruits and fruit juices and the risk of gestational diabetes mellitus: a prospective cohort study. *Diabetes Care* **2012**, *35*, 1079–1082, doi:10.2337/dc11-2105.

28. Savaiano, D.A.; Hutkins, R.W. Yogurt, cultured fermented milk, and health: a systematic review. *Nutr. Rev.* **2020**, *0*, 1–16, doi:10.1093/nutrit/nuaa013.

29. Asemi, Z.; Samimi, M.; Tabasi, Z.; Talebian, P.; Azarbad, Z.; Hydarzadeh, Z.; Esmaillzadeh, A. Effect of daily consumption of probiotic yoghurt on insulin resistance in pregnant women: A randomized controlled trial. *Eur. J. Clin. Nutr.* **2013**, *67*, 71–74.

30. Pellonperä, O.; Mokkala, K.; Houttu, N.; Vahlberg, T.; Koivuniemi, E.; Tertti, K.; Rönnemaa, T.; Laitinen, K. Efficacy of fish oil and/or probiotic intervention on the incidence of gestational diabetes mellitus in an at-risk group of overweight and obese women: A randomized, placebo-controlled, double-blind clinical trial. *Diabetes Care* **2019**, *42*, 1009–1017, doi:10.2337/dc18-2591.

31. Bakker, R.; Steegers, E.A.P.; Obradov, A.; Raat, H.; Hofman, A.; Jaddoe, V.W.V. Maternal caffeine intake from coffee and tea, fetal growth, and the risks of adverse birth outcomes: The Generation R Study. *Am. J. Clin. Nutr.*

2010, *91*, 1691–1698, doi:10.3945/ajcn.2009.28792.

32. Chen, L.W.; Fitzgerald, R.; Murrin, C.M.; Mehegan, J.; Kelleher, C.C.; Phillips, C.M. Associations of maternal caffeine intake with birth outcomes: Results from the Lifeways Cross Generation Cohort Study. *Am. J. Clin. Nutr.* **2018**, *108*, 1301–1308, doi:10.1093/ajcn/nqy219.
33. Van Der Hoeven, T.; Browne, J.L.; Uiterwaal, C.S.P.M.; Van Der Ent, C.K.; Grobbee, D.E.; Dalmeijer, G.W. Antenatal coffee and tea consumption and the effect on birth outcome and hypertensive pregnancy disorders. *PLoS One* **2017**, *12*, e0177619, doi:10.1371/journal.pone.0177619.
34. Sengpiel, V.; Elind, E.; Bacelis, J.; Nilsson, S.; Grove, J.; Myhre, R.; Haugen, M.; Meltzer, H.M.; Alexander, J.; Jacobsson, B.; et al. Maternal caffeine intake during pregnancy is associated with birth weight but not with gestational length: Results from a large prospective observational cohort study. *BMC Med.* **2013**, *11*, 42, doi:10.1186/1741-7015-11-42.
35. Skreden, M.; Bere, E.; Sagedal, L.R.; Vistad, I.; Overby, N.C. Changes in beverage consumption from pre-pregnancy to early pregnancy in the Norwegian Fit for Delivery study. *Public Health Nutr.* **2015**, *18*, 1187–1196, doi:10.1017/S136898001400189X.
36. Chen, L.W.; Low, Y.L.; Fok, D.; Han, W.M.; Chong, Y.S.; Gluckman, P.; Godfrey, K.; Kwek, K.; Saw, S.M.; Soh, S.E.; et al. Dietary changes during pregnancy and the postpartum period in Singaporean Chinese, Malay and Indian women: The GUSTO birth cohort study. *Public Health Nutr.* **2014**, *17*,

1939–1948, doi:10.1017/S1368980013001730.

37. Reis, C.E.G.; Dórea, J.G.; da Costa, T.H.M. Effects of coffee consumption on glucose metabolism: A systematic review of clinical trials. *J. Tradit. Complement. Med.* **2019**, *9*, 184–191, doi:10.1016/j.jtcme.2018.01.001.

38. Shi, X.; Xue, W.; Liang, S.; Zhao, J.; Zhang, X. Acute caffeine ingestion reduces insulin sensitivity in healthy subjects: a systematic review and meta-analysis. *Nutr. J.* **2016**, *15*, 103, doi:10.1186/s12937-016-0220-7.

39. Tunnicliffe, J.M.; Shearer, J. Coffee, glucose homeostasis, and insulin resistance: Physiological mechanisms and mediators. *Appl. Physiol. Nutr. Metab.* **2008**, *33*, 1290–300, doi:10.1139/H08-123.

40. Hinkle, S.N.; Laughon, S.K.; Catov, J.M.; Olsen, J.; Bech, B.H. First trimester coffee and tea intake and risk of gestational diabetes mellitus: A study within a national birth cohort. *BJOG An Int. J. Obstet. Gynaecol.* **2015**, *122*, 420–428, doi:10.1111/1471-0528.12930.

41. Adeney, K.L.; Williams, M.A.; Schiff, M.A.; Qiu, C.; Sorensen, T.K. Coffee consumption and the risk of gestational diabetes mellitus. *Acta Obstet. Gynecol. Scand.* **2007**, *86*, 161–166, doi:10.1080/00016340600994992.

42. Ministry of Health Malaysia *Malaysian Adult Nutrition Survey 2003. Habitual food intake of adults aged 18 to 59 years.*; Nutrition Section, Family Health Development Division, Ministry of Health, Malaysia: Putrajaya, Malaysia, 2008;
